# Combining Traits and Density to Model Recruitment of Sessile Organisms

**DOI:** 10.1371/journal.pone.0057849

**Published:** 2013-03-01

**Authors:** Luis Giménez, Stuart R. Jenkins

**Affiliations:** School of Ocean Science, Bangor University, Menai Bridge, United Kingdom; National Institute of Water & Atmospheric Research, New Zealand

## Abstract

We propose an integrative approach that explains patterns of recruitment to adult populations in sessile organisms by considering the numbers of individuals and their body size. A recruitment model, based on a small number of parameters, was developed for sessile organisms and tested using the barnacle *Semibalanus balanoides*, a marine invertebrate inhabiting North Atlantic intertidal shores. Incorporating barnacle body size improved model fit beyond that based on density alone, showing that growth played an important role in how resource limitation affected survival. Our approach uncovered the following: First, changes in the shape of the recruitment curve resulted from the balance between individual growth and mortality. Second, recruitment was limited by the least plastic trait used to characterise body size, operculum area. Basal area, a trait that responded to increases in barnacle density, did not contribute significantly to explain patterns of recruitment. Third, some temporal variation is explained by changes in the amount of space occupied by shells of dead barnacles: at high cover barnacles are densely packed and these shells remain long after death. Fourth, seasonal variation and spatial variation in survival can be separated from that resulting from resource limitation; survival was predicted for two different shores and four sampling times using a single recruitment model. We conclude that applying this integrative approach to recruitment will lead to a considerable advance in understanding patterns of mortality of early stages of sessile organisms.

## Introduction

Recruitment, the process by which young are incorporated into adult cohorts, is a key contributor to the structure and dynamics of populations. The input of new individuals to a population is followed by a critical period, generally of high mortality [1], during which patterns of adult abundance are established. To what extent spatial and temporal variation in the input of new individuals determines local adult dynamics has been experimentally tested and modelled extensively in marine systems [2], [3]. Pelagic dispersal of larval propagules in fish and the majority of benthic invertebrates [4] mean greater decoupling between local reproduction and input into local populations. Consequently, populations of benthic invertebrates with a larval phase are on average more demographically open [2], [5]. A body of research has focused on whether populations are ‘recruitment limited’ (sensu [6]) and hence structured by density-independent processes dictated by events occurring in the pre-settlement, larval phase or whether delivery of larvae saturates resources leading to a strong role for post-settlement density-dependent mortality (see [3] for review). Recent work on sessile benthic invertebrates suggests that variation in input of new individuals to benthic populations can have both positive and negative effects on adult abundance [7]. Present theory of recruitment in marine organisms and its impacts on community ecology has attempted to integrate processes occurring at all stages of the life cycles [8]. For instance Alexander and Roughgarden [9] modelled larval transport and predicted how populations are controlled by changes in settlement rates forced by upwelling conditions in the North Pacific Ocean. However one potentially important element which models of recruitment rarely address is variation in individual traits.

In most theories of recruitment, individuals are considered to be equal. However, there are several lines of evidence suggesting that much understanding can be gained if phenotypic variation among individuals is considered as a potential modifier of recruitment patterns. First, there is now a considerable body of work showing that variability in body size or physiological condition exhibited by larval stages can affect pre- and post-metamorphic survival [10], [11], [12], [13]. Given the high levels of mortality observed across wide ranging taxa during early juvenile life [1], one should expect variation in individual traits to impact patterns of recruitment. Second, as organisms grow they use more resources. Since growth rates in early stages can be very high (e.g. 50 fold increases in body mass within five weeks in crustacean larvae: [14]) the use of resources increase considerably. More robust bodies contribute to the maintenance of dominance in spite of reductions in density by mortality [15]. Third, through ontogeny, organisms can change in their adaptations to stress [16] or food requirements [17].

We propose a model that attempts to explain patterns of recruitment as a consequence of resource limitation by incorporating changes in body size through time. This model focuses on the “process of recruitment”, defined as the process by which early stages of organisms survive and finally recruit to ( = are incorporated into) the adult cohorts. The models are tested using data from Jenkins et al. [7] on settlement-recruitment relationships of a sessile invertebrate, the barnacle *Semibalanus balanoides*. In this species, space is a key resource and space competition affect patterns of adult densities.

Our primary objective was to test to what extent incorporation of traits describing body size through time could enhance the ability to predict patterns of recruitment based on input of new individuals. Secondly, given an improvement of the recruitment model, we aimed to determine whether predictive ability varied with the specific descriptor of body size.

## Methods

### Model Formulation

We developed a cohort model based on discrete difference equations describing changes in abundance of a cohort, within a generation. In the general formulation the model is as follows:
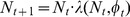
(1)where the number of individuals at time *t+1* is expressed as a function of the number at time, *t*, and a trait, *φ_t_* describing variations in body size through time. The function λ*(N_t_,φ_t_)* defines the fraction of individuals surviving to the next time step. The model starts with the number of settlers, *N_0_*. Predictions of the number of individuals reaching a given stage (e.g. adult), are achieved through iteration of eq.1.

The general formulation above resembles that of the neighbourhood models developed by Pacala and Silander [18], but we focused on survivorship from the juvenile to the adult stage, instead of considering the complete life cycle. This is not a stage-dependent model as those described in for example Caswell [19] and Zuidema et al. [20]: we wanted to avoid arbitrary definitions of stage categories when these are not naturally distinguishable. The model also differs from physiologically-structured models [21], [22] in that we do not model the dynamics of the individual traits and do not structure populations by body size categories. We model intra-cohort interactions, based on a small number of parameters and known trait values, assuming that individual traits can be captured by the average trait value of a cohort in a particular site and time. This is reasonable in the case of short-lived organisms where different cohorts may not interact. In addition, if the process of production of juveniles occurs over a very short time period, there is no overlap between cohorts of individuals of different ages. This assumption is valid for invertebrates which exhibit a single constrained period of breeding/larval release and subsequent pulse of settlement. The organism used here to test the model *S. balanoides*, shows such characteristics, releasing larvae over a short period in the early spring and settling over a relatively short period of one to two months [23].

As a first step we describe a simple mathematical expression linking body size and density: In the case of sessile species, as individuals grow, the amount of resources used at a given time depends on both density and body size. If the resource is space then the key variables may be the basal area or some other individual trait characterising the use of space. If the resource were food or nutrients, the key variable would be some function of the body mass of the recruits. In all these cases, survival must depend on the amount of resource, *A,* and the fraction of resources utilised by a population defined by the product *N_t_ ·*φ*_t_*. Since resource use cannot exceed available resources, we have: *A* ≥ *N_t_ ·*φ*_t_*. Therefore, the model of eqn. 1 becomes:
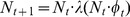
(2)


Next we describe the functional form of λ(N_t_,φ_t_): In many cases this form is derived by making assumptions about the distribution of individuals in space (e.g. [18], [24], [25]). However, derivation of the functional form in this way will depend on a number of factors, including the specific distribution patterns. We instead attempt to fit known functional forms to observed patterns of recruitment (e.g. see [26], [27]). This information could subsequently be used to guide the analytical derivation of a functional form.

We considered 3 functions: (a) negative exponential λ_t_ *= α•exp(−*β*•* φ*_t_ •N_t_)*; (b) logistic: λ_t_ *= *1/[1+α*’* exp(*−*β*•* φ*_t_ •N_t_* )]; (c) hyperbolic λ_t_ *= *α*/(1+*β •φ*_t_ •N_t_)*. These are all based on two parameters. Inserting these functions into eq. (1) leads to:
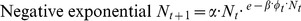
(3a)

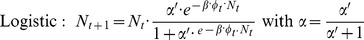
(3b)

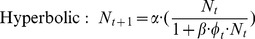
(3c)


In all models *α* is the resource-independent parameter. In the logistic model, the parameter *α* is calculated from another parameter *α’* (see [28] for the analogue case of the logistic population dynamics model). In addition, *β*, is the “resource use-dependent mortality parameter” and it has units of (individual size)^−1^; *1/*β is proportional to the carrying capacity of the system, i.e. to the total amount of available resources, *A*, at a given time and it has units of the resource (e.g. cm^2^ of surface area).

**Figure 1:**
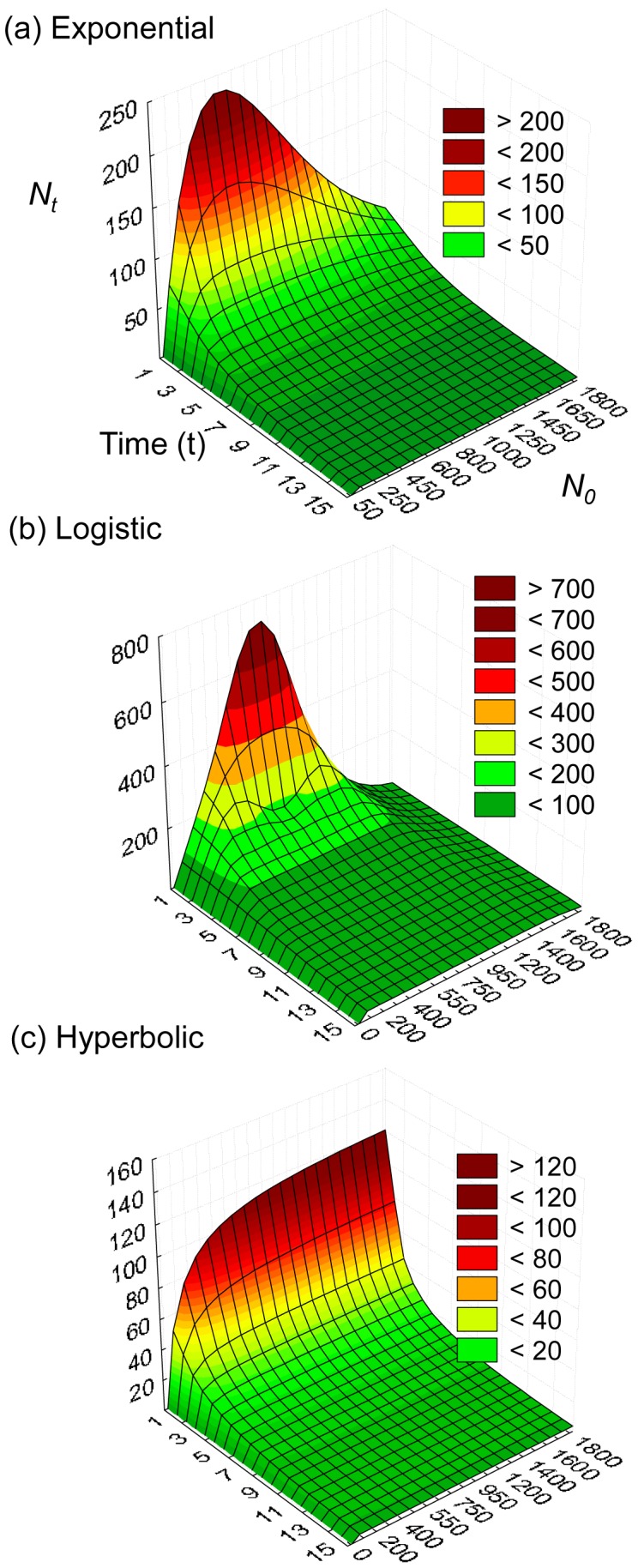
Maps of the recruitment function, i.e. *N_t+1_ = f(N_t_* φ*_t_)*, according to the different functional forms of survival for different initial abundance (*N_0_*) and time steps. These functions depend on the resource-independent parameter, *α* and the resource dependent parameter, *β*. To develop these maps, the body size, φ*_t_*, was modelled following a von Bertalanffy growth function, based on Ford-Walford model, φ_t+1 = _φ_t_ e^−K^+φ_∞_ (1-e^−K^), characterised by a growth rate (K = 0.01), and asymptotic body size (φ_∞_ = 1) and starting with an initial body size (φ_0_). Parameter values were as follows: for all panels, α = 0.99; other parameters (**a**) β = 0.5, φ_0_ = 0.01; (**b**) β = 0.2, φ_0_ = 0.01; (**c**) β = 0.1, φ_0_ = 0.1.

In the models (eqn 3a–c), the shape of the relationship between settlers and recruits (the recruitment function) varies through time according to the functional form used (Fig. 1). The exponential model (Fig. 1a) resembles the logistic population model [24], [25]; this function shows a maximum at some intermediate value of resource use and does not change as time progresses other than becoming progressively flatter. In our logistic recruitment model (Fig. 1b) the shape of the function does change: at an early time step there is a single point of compensation as in the exponential model, but in subsequent stages the curves have more than one maximum, i.e. there are 2 or more points of compensation. In the hyperbolic model (Fig. 1c) the curve increases monotonically, independent of the change in the parameter values.

### Model Application to Barnacle Recruitment

**Figure 2:**
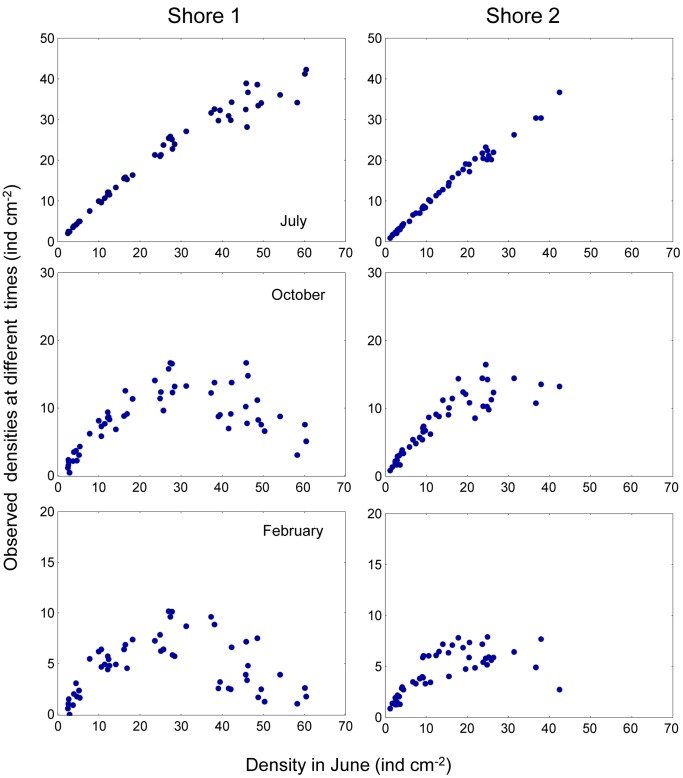
*Semibalanus balanoides*. Observed densities of juvenile barnacles for July, October 2002 and February 2003 for each site and shore.

*Semibalanus balanoides* is an intertidal sessile barnacle, abundant on exposed and semi-sheltered rocky shores of the Atlantic coast of North America and North Europe. This species is a pulse recruiter: cyprid larvae settle from the plankton and metamorphose during a short period (4–6-weeks) in spring [23]. Density-dependent processes are main contributors to patterns of mortality in this species. Jenkins et al. [7] studied recruitment patterns of *S. balanoides* at two different rocky intertidal sites in North Wales, UK. The authors manipulated the density of settling barnacles and made photographic observations of a single cohort of *S. balanoides* over two years. Jenkins et al. [7] showed that the magnitude of density-dependence varied through space and time, in response to variations in the density of settlers. The results corresponding to the first year of the study are shown in figure 2. In particular, a major finding in this work is that on both shores the shape of the settler-recruit curves changed from a monotonous increasing pattern in July (Fig. 2 upper panels) to one with over compensation in October and February (Fig. 2 middle and lower panels) that was maintained over the following year [7].

In order to evaluate the integrative models described in the previous section, we revisited the photographs of the quadrats (52 units 5×5 cm per shore) used to estimate barnacle density. Average basal area, *B_t_*, and operculum area, *O_t_*, (both in mm^−2^) per individual barnacle were estimated from the digital images based on 20 individuals per image. These were selected at random by overlaying 20 points on each photograph. Density data (Fig. 2) were taken from Jenkins et al. [7].

It is important to note that each quadrat was clear of all adults before the settlement period [7]; therefore, variations in density or cover respond entirely to variations in survival of juveniles that settled at the start of the experimental period.

One of the requirements of the model was that time periods between observations should be equal. Jenkins et al. [7] however made their observations at variable time intervals. To reduce the effect of variable time periods we restricted our model fitting to the first year of data when differences in time intervals were shorter. In addition, model fitting was undertaken for equal periods ( = 30 days) and then predictions were rescaled to the original time periods (see details in appendix S1). Models were adjusted by linear regression on the linearly transformed version of the models.

Model fitting depends on (1) whether traits are necessary to explain survival patterns and (2) if there is a particular functional form that better describes relationships between survival and resource use. We compared the fitting of the different functional forms with and without traits through the adjusted R^2^ and by inspection of the residuals. The best model should have the highest R^2^ but also residuals should not show any trend. If traits were important, then models incorporating any one of the traits should perform better than models that do not incorporate any trait.

Results showed important temporal variation in survival that was not related with space use. In order to incorporate this variation, we developed a general model where the resource independent parameter, *α*, was allowed to vary among sampling times. In addition, inspection of photographs showed that in October, shells of dead barnacles were present in several plots characterised by high barnacle densities. Although these were not considered as living individuals for the estimation of density, they might have contributed to temporal variation in barnacle survival. We therefore incorporated the effect of cover of shells of dead animals in order to evaluate their differential contribution to the total mortality. We used general linear modelling with model selection based on AIC (Akaike Information Criteria) since this allowed the comparison of models with different numbers of parameters.

## Results

### Density alone vs. Density *and* Traits as Predictors

The best models were those incorporating the operculum surface area as a descriptor of body size. These models showed highest R^2^ values (Table 1) for both shores and for all functional forms considered here; in particular they performed better than models based on density alone. From this set, the best functional form was the logistic: this resulted in the highest *R^2^* and best distribution of errors in comparison with other models (Table 1 and see appendix S2 for explanation and interpretation of model fitting errors). Both the exponential and hyperbolic forms showed non-linear patterns and lack of an even distribution in errors especially on Shore-1, indicating that these forms do not correctly explain survival over the whole range of resource use. Therefore, barnacle recruitment was best explained by combining operculum area and density as descriptors and using the logistic functional form.

**Table 1:** Barnacle recruitment.

	Exponential	Logistic	Hyperbolic
Shore-1			
Density	0.297	0.259	0.274
Density+Basal area	0.097	0.158	0.080
Density+operculum area	0.306	**0.312**	0.274
Shore-2			
Density	0.103	0.129	0.108
Density+Basal area	0.036	0.068	0.035
Density+operculum area	0.201	**0.214**	0.180

Adjusted R^2^ coefficients for functional forms (exponential, logistic and hyperbolic) in models with and without barnacle traits characterising body size (basal or operculum surface area). All models were significant (*P*<10^−3^).

**Figure 3:**
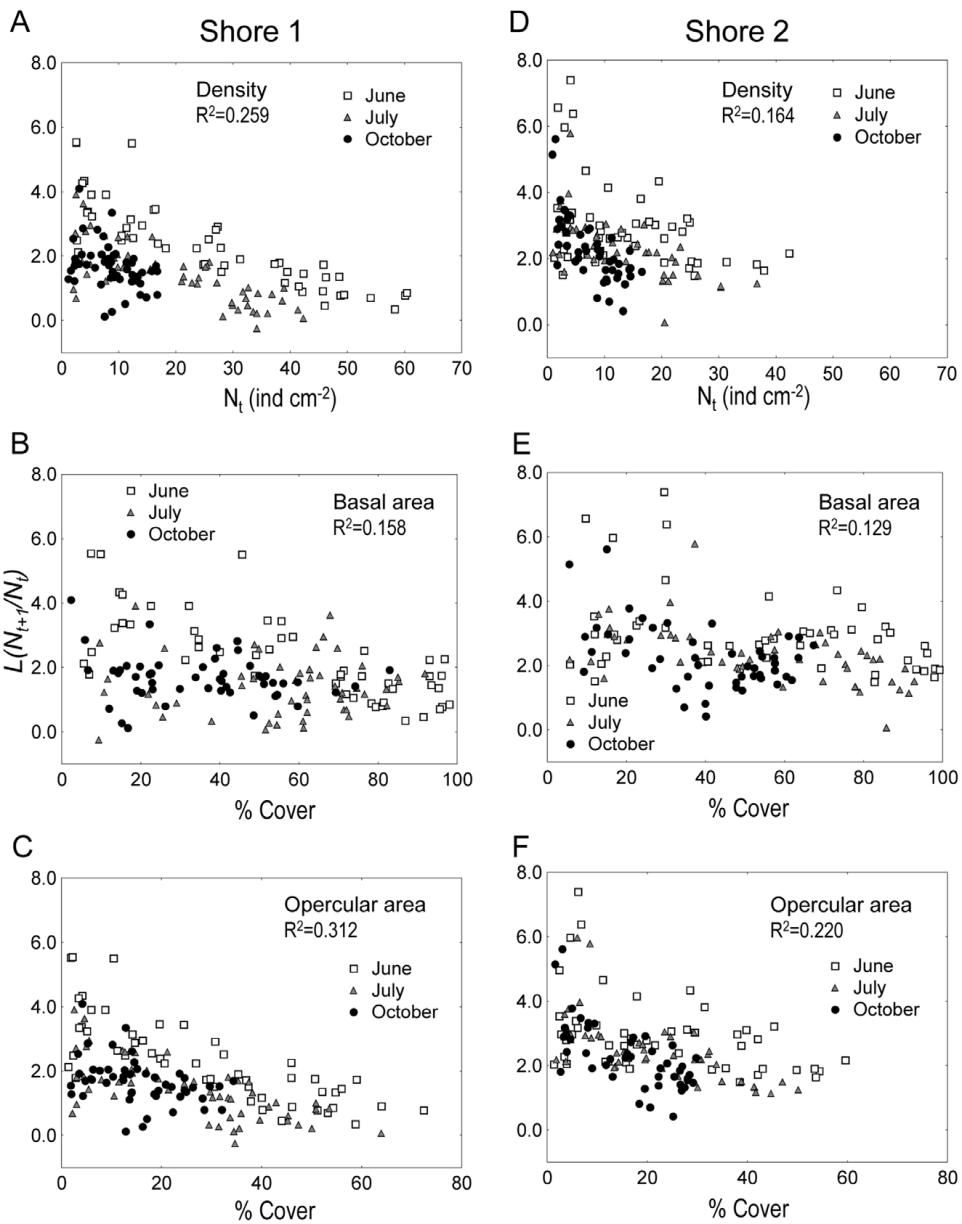
*Semibalanus balanoides*. Relationships between three descriptors of resource use and the logistic functional form (equation 3b in text, *Y_t_* = *N_t+1_/N_t_*). The descriptors of resource use were density *( = N_t_*), or cover based on basal area and cover based on operculum area. Symbols identify the 52 sites per shore according to the observation period: these are June-July (indicated as “June”), July-October (“July”) and October-February (“October”).

Figure 3 illustrates graphically the importance of incorporating operculum size in the model using the logistic functional form: models based on density alone or incorporating basal area did not explained well patterns of survival found between October and February. For that period (black symbols in Fig. 3), data points were scattered along the Y-axis when the descriptor was the barnacle density, even though barnacle density was low. By contrast, incorporating operculum area resulted in a better fit in all time periods: the variation in survival responded to variations in barnacle cover, calculated as the product of density and operculum surface area.

**Figure 4:**
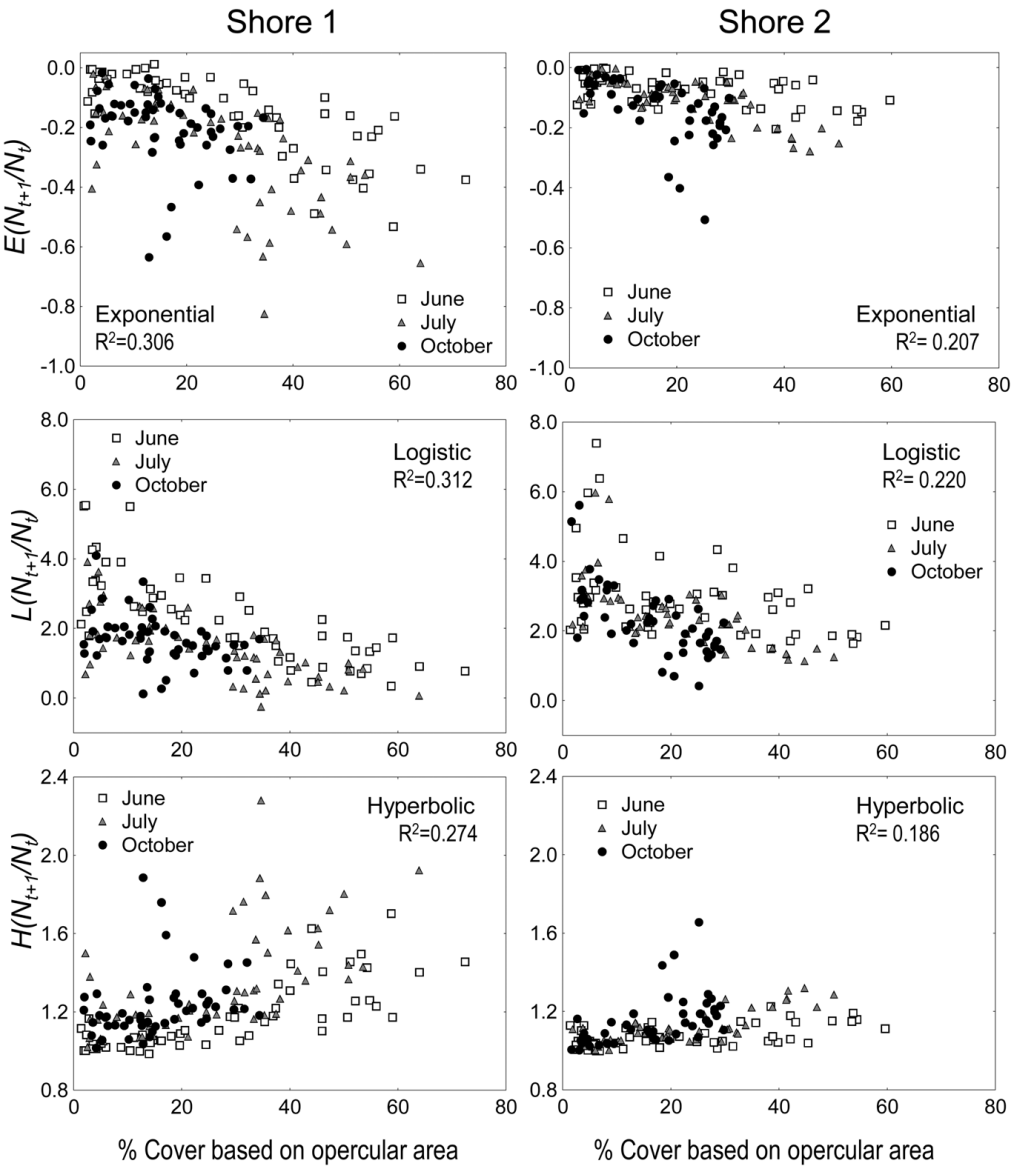
*Semibalanus balanoides*. Relationships between three functional forms of juvenile survival probabilities and a descriptor of resource use in two shores. The functional forms are plotted in their linear versions where *Y_t_* = *N_t+1_/N_t_*. Symbols identify the 52 sites per shore according to the observation period: these are June-July (indicated as “June”), July-October (“July”) and October-February (“October”). The plotted descriptor of resource use was cover, based on the product of the average operculum surface area and density.

Figure 4 illustrates graphically the importance of the logistic functional forms. Irrespective of the trait descriptor both the exponential and hyperbolic models showed a high level of dispersion towards values of high cover, while the logistic form resulted in homogeneous levels of dispersion (see also appendix S2 for error distribution).

**Figure 5:**
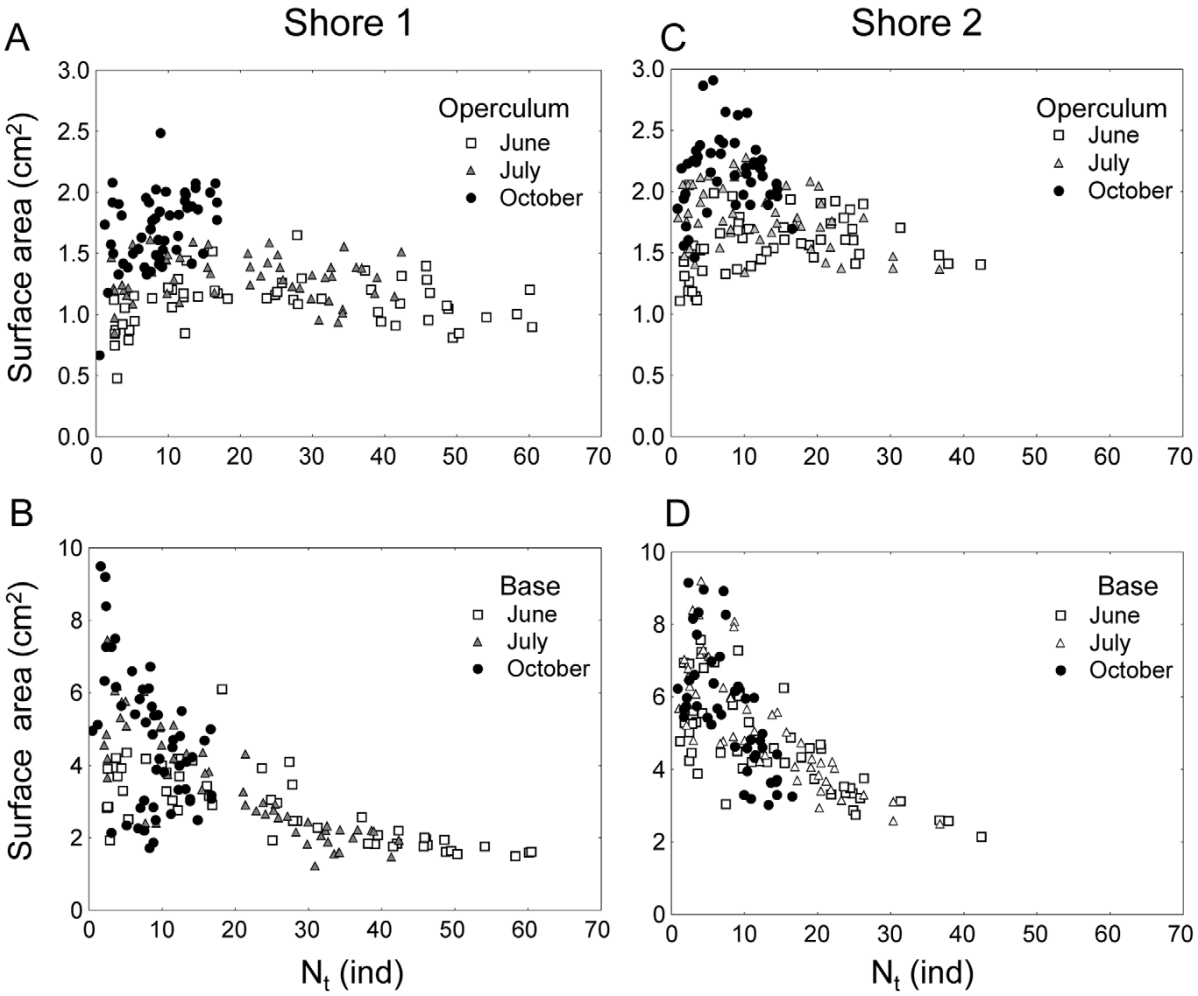
*Semibalanus balanoides*. Relationships between barnacle individual traits (average operculum and basal surface area) and density in 52 sites per shores and at three times (June, July, October).

In considering the better predictive power of models using operculum area as opposed to basal area, the way in which these traits respond to density is important. Operculum area showed low and non-significant correlation with density (Fig. 5a, c) while basal area responded plastically to density with lower average basal area per individual at higher barnacle density (Fig. 5b, d).

### Incorporating Temporal Variation in Survival

In this step, we evaluated if temporal variation in survival could be modeled as variations in the resource independent parameter, α, and variation in the cover of barnacles. The best model (lower AIC: appendix S3) incorporated both sources of variation. This is also seen in an increase in adjusted *R^2^* values (Shore 1: R^2^ = 0.57; Shore 2: R^2^ = 0.37) in comparison with models not incorporating this temporal variation (Shore 1: R^2^ = 0.31; Shore 2: R^2^ = 0.21: Table 1). The parameter *α* was higher for June-July than July-October and October-February (Shore 1: α_june-july_ = 0.97 α_july-october_ = 0.92; Shore 2: α_june-july_ = 0.98; α_july-october_ = 0.96). In addition, the influences of living vs. dead individuals differed according to the shore: On shore 1 there was a stronger effect of live animals on survival (β_live_ = 4.08•10^−2^, β_dead_ = 3.61•10^−2^) but the opposite pattern was found on Shore 2 (β_live_ = 3.21•10^−2^, β_dead_ = 8.36•10^−2^).

**Figure 6:**
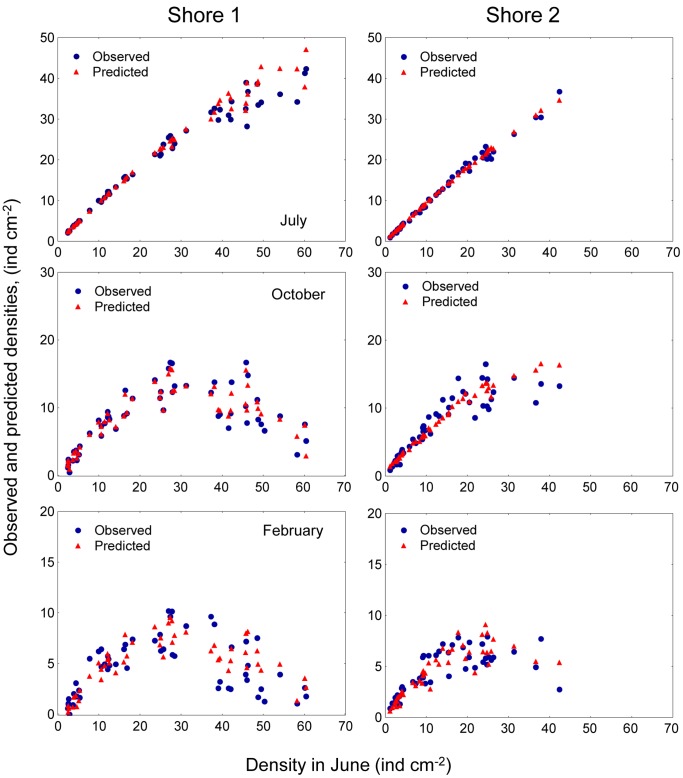
*Semibalanus balanoides*. Model predictions and observed densities of juvenile barnacles for July, October 2002 and February 2003 for each site and shore.

This final model, which incorporated temporal variation, captured the change in the form of the recruitment function from a monotonic increasing pattern for densities in July, to a convex pattern for densities in October and February (Fig. 6). In addition, the model captured some of the variation along the y-axis displayed by the original data, suggesting that this was due to variations in the barnacle operculum area in combination with variations in the amount of space occupied by shells of dead individuals. In summary, models showed that barnacle recruitment depended on changes in cover, governed by reductions in density and increases in operculum size, and also by temporal variation in resource-independent survival and by the amount of space used by shells of dead barnacles. Strong resource-dependent mortality occurred in populations with high initial densities and large initial body size: these two factors limited the use of space.

## Discussion

Here we show that integrative recruitment models can quantify previously known but not quantified processes affecting recruitment as well as uncovering a series of mechanisms that explain patterns of juvenile survival and ultimately the number of individuals recruiting to adult stages. These mechanisms cannot be derived from models based solely on variations in density of individuals. We show how model selection can be used to statistically infer important traits and critical trait values involved in density-dependent survival. Our model fitting exercise using *Semibalanus balanoides* allowed us to quantify how recruitment responds to the combination of body size and density. Body size should affect recruitment through resource limitation. Resource limitation, modelled by the product of individual body size and density explained why the settlement-recruit curve changed from a monotonic increasing pattern, observed in July, to a pattern with a point of compensation observed after October [7]. These changes can now be understood as a result of the balance between individual growth and mortality in combination with a logistic functional response. Our study highlighted the role of trait plasticity in explaining recruitment: the key trait, (operculum size) was the one with limited or no plastic response to barnacle density. In addition our statistical approach allowed us to separate variation due to resource use from variations due to resource-independent mortality (variations in *α*).

### The Importance of Body Size for Temporal Changes in Mortality

A major feature in the process of recruitment of *S. balanoides* is the temporal change in the patterns of mortality at high densities, reflected in the change in the recruitment curve from a monotonic increasing pattern to a pattern characterized by overcompensation [7]. Our study offers a biological explanation for that change in animal growth and quantifies the effect of growth on recruitment. We did not estimate growth rates in the field because model fitting does not require knowledge of a growth curve; our models require direct knowledge of body sizes at each time interval. Variations in body size must obviously reflect variations in post-settlement growth.

Irrespective of the functional form, models based on density alone cannot explain the change in the type of recruitment curve from monotonous increasing to overcompensation. Simulations (appendix S4) show that recruitment models do not predict changes in the shape of the settlement-recruitment curve. Such change responds to the combined effect of body size and density incorporated in a logistic functional response. Most likely, changes in body size and variations in density of individuals control the amount of free space: this may decrease, increase or remain constant according to the balance between individual growth rates and mortality rates. In our case, free space remained constant over the first month suggesting that individual growth matched mortality. Once space use reached a critical level of 30–40%, mortality dominated over individual growth.

### Spatial Patterns in Mortality and the Importance of the Functional Form

The logistic form was the functional form that best explained spatial patterns of survival. The functional forms should reflect the way organisms respond to space limitation, but there is little information about the biological processes underlying the logistic or any similar form. Responses with inflexion points such as the logistic or the sigmoid are known and are expected in animals producing a large number of offspring depleting the resources [26].

A way to attempt to determine the mechanisms underlying the logistic response is to evaluate assumptions made in the derivation of other responses. Many models derive the exponential form; this is done under the assumptions that (1) organisms distribute following Poisson and (2) survival chances decrease geometrically with the density of neighbors [18], [24]. Thus, deviations from the exponential functional form may reflect violation of one or both assumptions. For example higher than expected mortalities found at high densities may be explained if barnacles aggregate more at these densities than at low density. In addition, in our case, space use depends also on the distribution of body sizes. Inspection of our photographs suggests that barnacles aggregate at low densities and that space is occupied evenly at high densities. In addition, variation in densities, not in body size, was the main contributor to the spatial variation in resource use (body size affected temporal variation in recruitment). Therefore, variations in the distribution in response to average densities should not be responsible for the observed deviations from the exponential functional form.

A second explanation is that individual survival chances do not respond geometrically to local density. Instead, mortality could reflect some type of threshold phenomena operating at the individual level. Model simulations (see Appendix S5) suggest that the recruitment curves follow a pattern that resembles the one observed for *S. balanoides* if: (1) individual survival follows a threshold response to density, (2) resource use depends on growing organisms and (3) individuals distribute as Poisson. Therefore, threshold phenomena in response to density may underlie the chances of survival in *S. balanoides*. These threshold phenomena may be related for instance to the minimum number of neighbors surrounding a barnacle that are necessary to break its shell or to the minimum number of individuals that are necessary to produce hummock structures; these structures increase chances of organisms being irreversibly removed from the habitat by waves.

### Trait Plasticity and Recruitment

The trait that explained patterns of barnacle survival, opercular area, was the least plastic. An adaptive phenotype can buffer the performance of organisms from variation in local conditions [29], [30]. In barnacles, morphology and behavior respond plastically to food availability, crowding, wave exposure or predation [31], [32], [33]. Reduced body size in response to increases in density of conspecifics is common in plants and animals [15], [34] and in barnacles in particular [35], [36], [37]. However, not all traits are equally plastic and trait plasticity must respond to physiological constraints and selective forces. In *S. balanoides*, trait plasticity consists of a reduced basal area in response to barnacle density. As barnacles grow, those at high densities are constrained from expanding the basal area, but continue to thrive (usually by growing upwards) as long as the basal area allows sufficient strength to attach in the face of wave forces. By contrast, opercular area appears to behave less plastically presumably because this trait will act as a limiting factor in accessing oxygen and food.

Shells of dead organisms can create or modify microhabitat [38], [39], and increase recruitment [40] but in our case, shells resulted in reductions in resource-dependent survival at high barnacle cover. Barnacle shells may reduce space available for food capture or exert mechanical forces on the exoskeleton of the live individuals, thereby contributing to their breakage. At high densities, shells still contribute to the formation of hummock structures. More generally, this type of effect may be important in systems characterized by low turnover rate of key resources.

Variation among shores and temporal variations in survival, captured as changes in the resource independent parameter (α), are likely to occur in *S. balanoides* [41], [42], [43] and other barnacles [44], as a consequence of temporal and spatial variation in abiotic factors, food or physiological conditions of juveniles. We do not attempt to explain these variations here, but we conclude that our modeling approach will help to understand the causes of these variations if applied to a larger number of sites and sampling times.

### Perspectives

Most approaches to understand population and community dynamics make the assumption that patterns of reproduction, mortality and migration provide sufficient information to understand changes in abundance of organisms. However, there is a growing body of literature showing that a considerable advance can be made if development and individual growth are also considered [17], [21], [22], [45]. Our work is consistent with this view. For instance, Claessen et al. [17], [21] showed that ontogenetic changes in diet and in competitive abilities affect population dynamics and size distribution in fish. We show that the interplay of growth and density determines the level of competition and so the settler-recruit relationship. In future work the incorporation of growth and development into population level studies appears essential to advance our understanding of population dynamics.

In the specific case of marine species with complex life cycles, integrative framework offers a way to link different types of pre and post-settlement processes that shape recruitment. We know that larval behaviour [46] and supply affect settlement rates [2], [9], which in turn co-determine the magnitude of biotic interactions [47]. We also know that larval phenotype can affect settlement patterns and that larval physiological quality can affect post-metamorphic performance (barnacles: [12], [48], [49]; other invertebrates [11], [50]). However, only recently have we seen that recruitment may depend on both numbers and phenotypes of individuals (e.g. [13], this study). The interplay of growth and density should explain recruitment in many species where intra-specific competition is documented ([51], [52], [53], [54]). Thus, integrative models will provide a way to study how the interplay of growth and density affects the chances of organisms reaching the reproductive stage.

## Supporting Information

Appendix S1 **Data analysis for barnacle recruitment.**
(PDF)

Appendix S2 **Distribution of errors after model fitting of barnacle recruitment.**
(PDF)

Appendix S3 **Results of model building by Akaike Information Criteria.**
(PDF)

Appendix S4 **Predictions based on models with and without body size.**
(PDF)

Appendix S5 **A model simulation for threshold survival.**
(PDF)
