# Supplementary material for: Combining Traits and Density to Model Recruitment of Sessile Organisms
Source: PLoS One. 2013 Mar 1;8(3):e57849. doi: 10.1371/journal.pone.0057849 (PMC3585730; doi:10.1371/journal.pone.0057849)
Supplement: Appendix S1 — Data analysis for barnacle recruitment. (PDF) [file pone.0057849.s001.pdf]

Appendix S1- Barnacle recruitment-methods

One of the requirements of the model was that time periods between observations should be equal. Jenkins et al. (2008) however made their observations at variable time intervals. To reduce the effect of variable time periods we restricted our model fitting to the first year of data when differences in time intervals were shorter. In addition, predictions were made through three main steps (Fig S1):

(1) Values of were estimated at intervals of 1 month (=30 days) from observed values (N_t) by means of a cubic spline. The interpolated values, denoted as "N_{t+1}" correspond to those that would be expected one month after the observations took place; these values retained the patterns observed in the original data (Fig. S2, Table S1). The use of the splines ensured that the estimation of N_{t+1} was not based in any particular assumption.

(2) Model fitting was done using observed densities (N_t), traits (O_t and B_t) in June, July and October and using the interpolated densities, N_{t+1} corresponding to each of these observations. The model predictions (P_{t+1}) are therefore predicting N_{t+1} values.

(3) For the best model, predictions (P_{t+1}) were adjusted (P_{t+k}) in order to predict observations. For instance predictions made with densities of July as N_t and their respective N_{t+1} values were adjusted to predict densities in October. This procedure was made through linear equations obtained by regressing N_{t+1} values (independent variable) and the subsequent observation, $N_{t+k,}$ (response variable). Parameters to adjust model predictions are given in Table A2. These equations simply translate the position of a point in a space defined by $N_{t+1}=f(N_t)$ to a space defined by $N_{t+k}=f(N_t)$ without affecting model fitting (done in step 2).

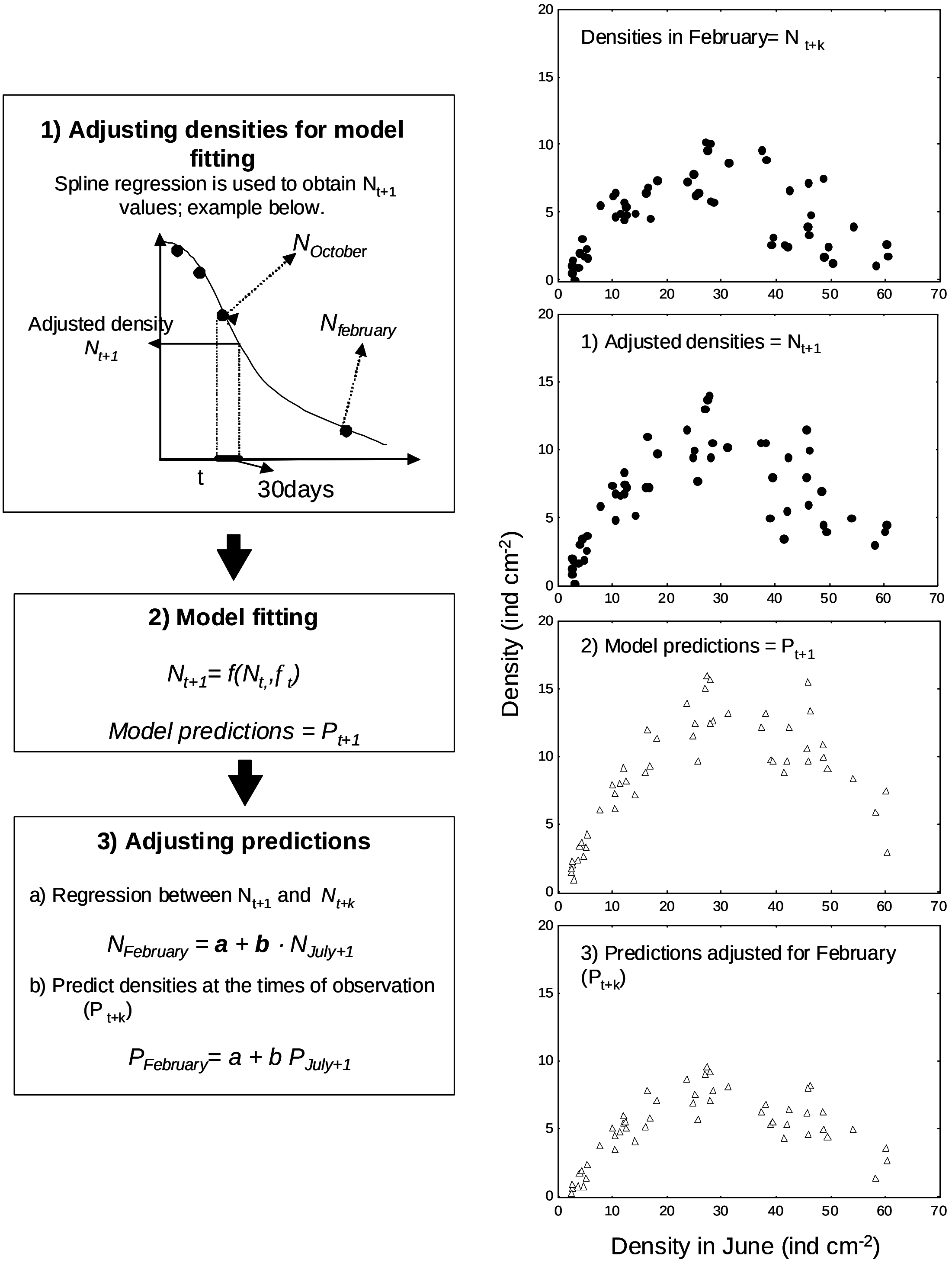

Figure S1. Summary of data treatment with an example for February 2003 (right panels). There were three steps: (1) for each site a cubic spline function was adjusted to the observed densities and values were interpolated at a regular time interval of one month (=30 days) after each observation these are called "N_{t+1}",. (2) Values of N_{t+1} and observations of density (N_t) and traits (φ_t) were used to fit models, $N_{t+1} = f(N_t,\varphi_t)$, predicting barnacle densities at 1 month after each observation (P_{t+1}). (3) Predictions of barnacle density (P_{t+k}) at the time of observation were made through an extrapolation procedure explained in the text.

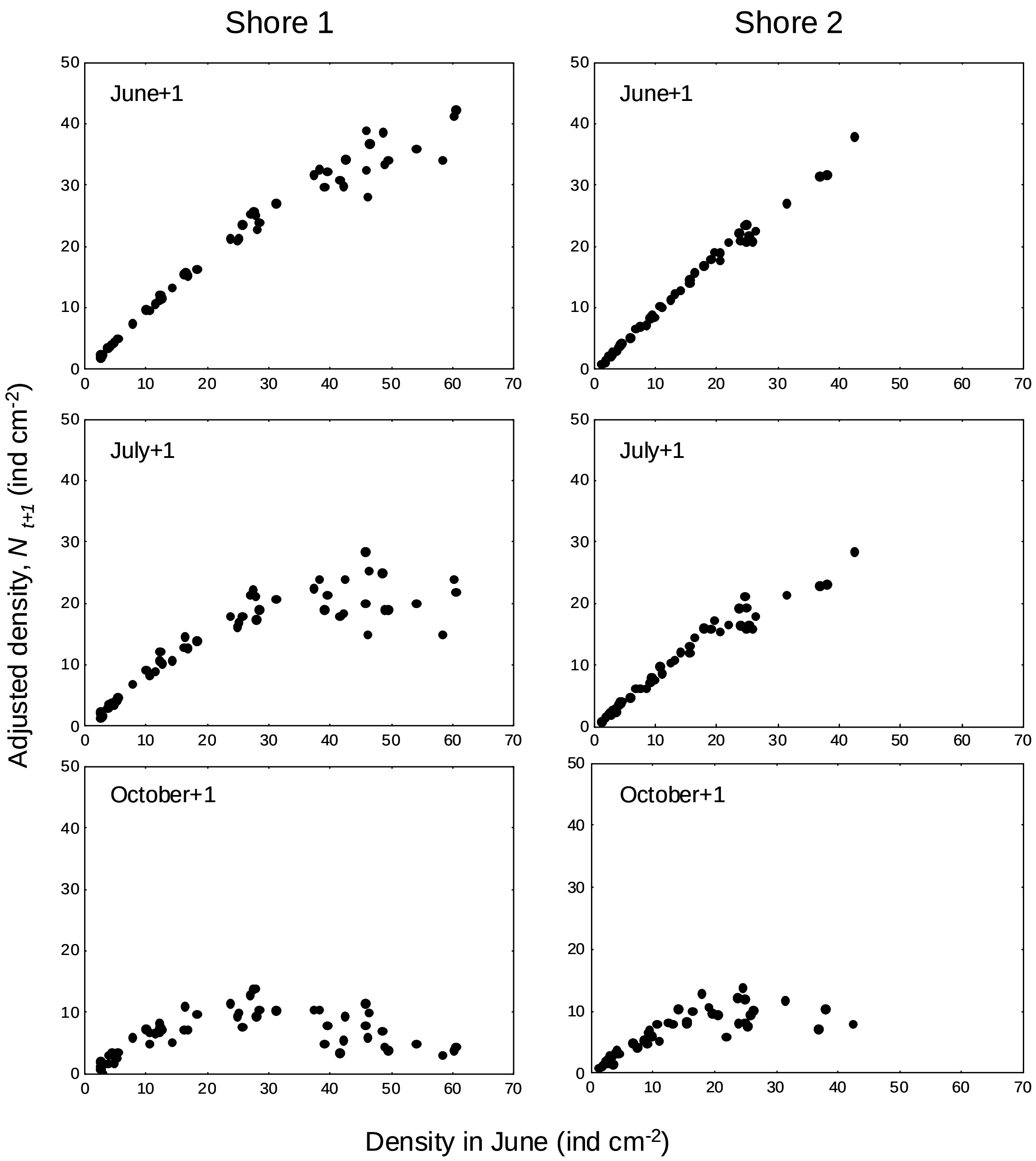

Figure S2. *Semibalanus balanoides*. Relationships between estimated densities (N_{t+1}) and initial densities (N_0) to study recruitment in two shores. The density estimates (N_{t+1}) were obtained by interpolation using spline functions adjusted to times series of densities collected in June, July, October-2002 and February-2003. Interpolated values correspond to the expected densities 30 days after the observations made in June, July and October. Each of the 52 points plotted here is a site located within a respective shore.

Table S1. Models and parameters of equations used to estimate densities at the time of observation

Model	*a*	*b*	*c*	R^2
Shore-1				
$N_{July} = a + b \cdot N_{June+1}$	-0.001	1.000		0.999
$N_{October}\ a + b \cdot N_{July+1} + c \cdot (N_{July+1})^2$	0.552	0.749	-0.010	0.683
$N_{February} = a + b \cdot N_{October+1}$	1.195	1.172		0.838
Shore-2				
$N_{July} = a + b \cdot N_{June+1}$	-0.187	1.038		0.999
$N_{October}\ a + b \cdot N_{July+1}$	-1.254	1.536		0.881
$N_{February} = a + b \cdot N_{October+1}$	-0.149	1.526		0.831

Note: The densities predicted for October were further adjusted by the residual of the polynomial regression. Therefore, the equation used to link observations made in October and the estimated density N_{t+1} between July and October was

Observed $N_{October,site\ j} = \boldsymbol{a} + \boldsymbol{b} \cdot Estimated\ N_{July+1,site\ j} + c \cdot (Estimated\ N_{July+1,site\ j})^2 + \textbf{residual}_{\textbf{site j}}$

so that the densities at the time of each observation were predicted using the parameters *a*, *b* and the *residuals* of the equation above as:

Predicted $N_{October\ i,site\ j} = \boldsymbol{a} + \boldsymbol{b} \cdot Predicted\ N_{July+1,site\ j} + c \cdot (Predicted\ N_{july+1,site\ j})^2 + \textbf{residual}_{\textbf{site j}}$
