# Supplementary material for: Combining Traits and Density to Model Recruitment of Sessile Organisms
Source: PLoS One. 2013 Mar 1;8(3):e57849. doi: 10.1371/journal.pone.0057849 (PMC3585730; doi:10.1371/journal.pone.0057849)
Supplement: Appendix S2 — Distribution of errors after model fitting of barnacle recruitment. (PDF) [file pone.0057849.s002.pdf]

Appendix S2-Model fitting: errors

The figures below summarise the distribution of errors of models with two parameters adjusted to survival of *Semibalanus balanoides* in two intertidal shores using the exponential, logistic and hyperbolic functional forms. Panels show errors for different independent variables: Figure S3: barnacle density; Figure S4 cover estimated from density and average opercular area.

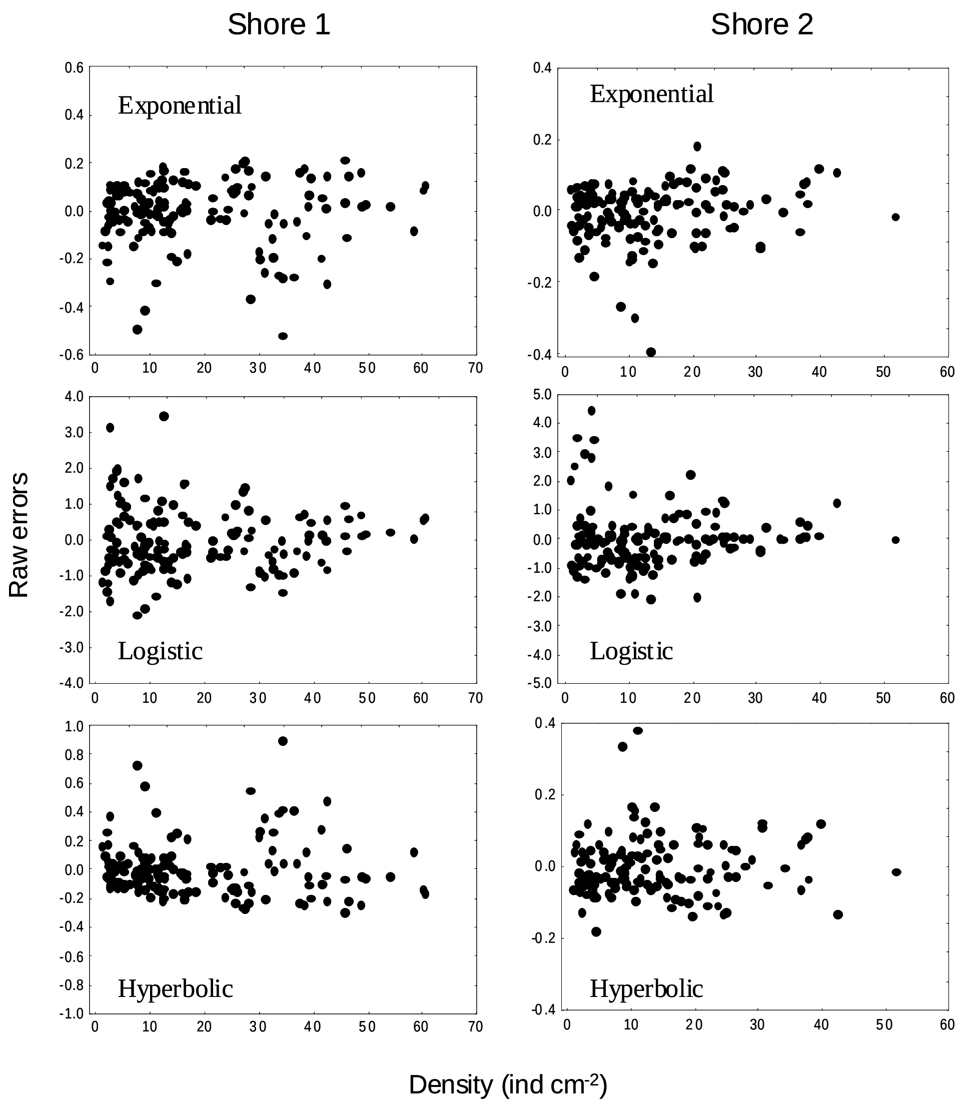

Figure S3. Distribution of errors for models based on density

Errors showed the same patterns irrespective of the independent variable but varied mainly according to the functional form of survival. Errors from the exponential and hyperbolic functional forms show trends and are not evenly distributed; the amount of dispersion at densities, 30 ind cm^{-2} or cover <40% is lower than that beyond these values. Errors from the logistic functional forms did not show trends and they were more evenly distributed. Panels corresponding to regressions with other independent variables show similar patterns as these two groups.

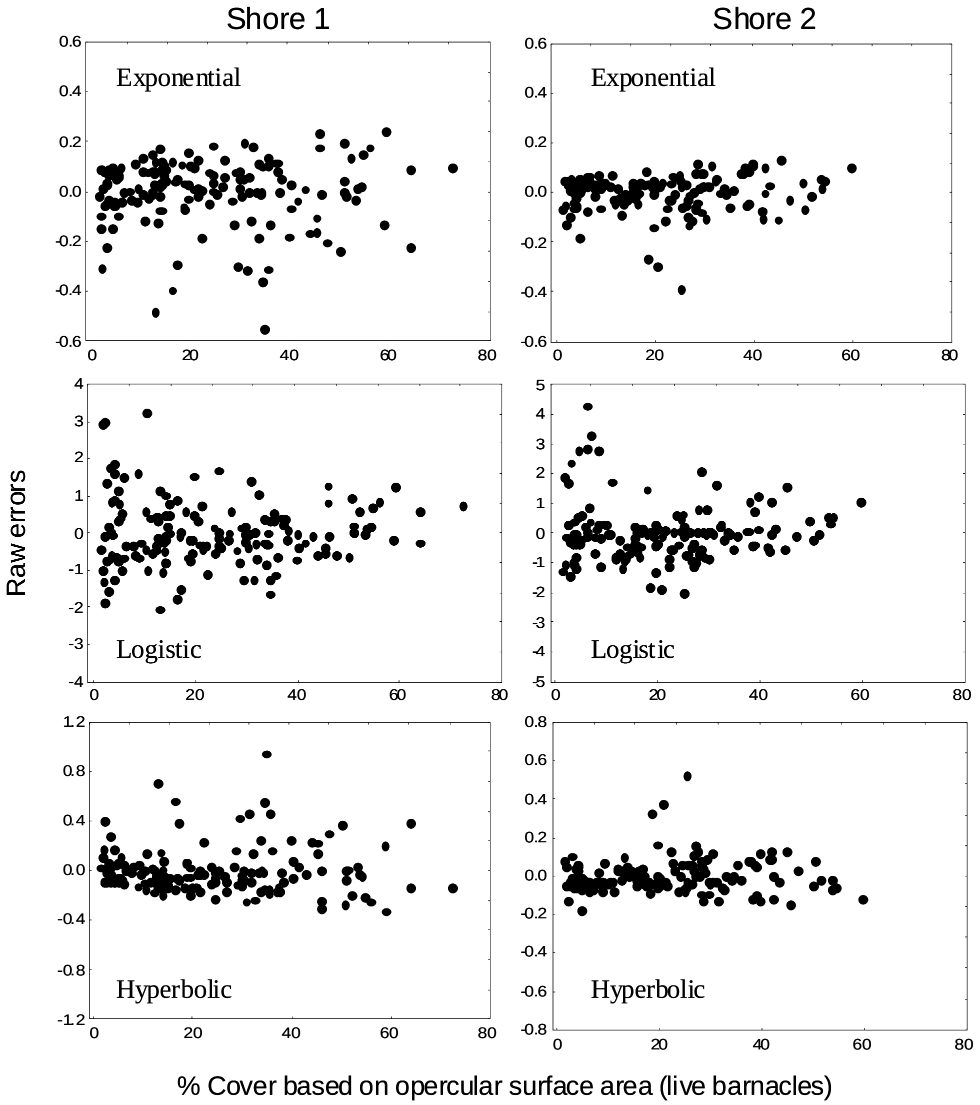

Figure S4. Distribution of errors for models based on density and operculum area.
