# Supplementary material for: Combining Traits and Density to Model Recruitment of Sessile Organisms
Source: PLoS One. 2013 Mar 1;8(3):e57849. doi: 10.1371/journal.pone.0057849 (PMC3585730; doi:10.1371/journal.pone.0057849)
Supplement: Appendix S3 — Results of model building by Akaike Information Criteria. (PDF) [file pone.0057849.s003.pdf]

Appendix S3 - Results of model building by Akaike Information Criteria

The table S2 summarises of results on general regression model building using Akaike Information Criteria and Likelihood ratios to fit the logistic functional form of survival probabilities of *Semibalanus balanoides* in two intertidal shores. The factors of the model tested were the resource independent survival probability, α' (allowed to vary among time intervals) and the cover of live and dead barnacles based on opercular area (X_1 and X_2: respectively) according the equation below ($Y_t = N_{t+1}/N_t$).

$$\ln\left(\frac{Y_t}{1-Y_t}\right) = \ln(\alpha')_\tau - \beta_1 \cdot X_{1,t} - \beta_2 \cdot X_{2,t}$$

Table S2. Abbreviations: *Ti*: factor accounting for variation in resource independent survival probability among time intervals; *df*: degrees of freedom; *AIC*: Akaike coefficient; *ΔAIC*: differences between consecutive in *AIC* values; *LR*: Likelihood ratios. All models were significant (P<0.001).

Shore 1							Shore 2						
Factors			*df*	*AIC*	*ΔAIC*	*LR*	Factors			*df*	*AIC*	*ΔAIC*	*LR*
X_1	X_2	*Ti*	4	331.51	6.57	132.42	X_1	X_2	*Ti*	4	364.27	12.43	73.11
X_1		*Ti*	3	338.07	30.92	123.86	X_1		*Ti*	3	376.70	2.61	58.68
X_1	X_2		2	368.99	30.62	90.94	X_1	X_2		2	379.32	12.80	54.07
X_1			1	399.61	15.51	58.31		X_2	*Ti*	3	392.12	5.15	43.26
	X_2	*Ti*	3	415.12	13.23	46.81	X_1			1	397.27	5.02	34.12
	X_2		1	428.35	10.59	29.57		X_2		1	402.28	18.39	29.10
		Ti	2	438.95		20.98			*Ti*	2	420.68		12.71

.
