# Supplementary material for: Combining Traits and Density to Model Recruitment of Sessile Organisms
Source: PLoS One. 2013 Mar 1;8(3):e57849. doi: 10.1371/journal.pone.0057849 (PMC3585730; doi:10.1371/journal.pone.0057849)
Supplement: Appendix S4 — Predictions based on models with and without body size. (PDF) [file pone.0057849.s004.pdf]

Appendix S4 - Predictions based on models with and without body size

Models based on density

Models without body size reduce to:

Negative exponential: $N_{t+1} = \alpha \cdot N_t \cdot e^{-\beta \cdot N_t}$

Logistic: $N_{t+1} = N_t \cdot \frac{\alpha' \cdot e^{-\beta \cdot N_t}}{1+\alpha' \cdot e^{-\beta \cdot N_t}}$ with $\alpha = \frac{\alpha'}{\alpha'+1}$

Hyperbolic: $N_{t+1} = \alpha \cdot (\frac{N_t}{1+\beta \cdot N_t})$

The maps of the different models are shown below (Fig. S5 left panels). Exponential and logistic models show overcompensation at high densities. Over several time steps, the general form of the recruitment function is preserved. In particular, the compensation points are already established at earliest time step and remain constant with respect to the initial densities, at all time steps.

Models based on density and body size

Right panels in Figure S5 simulate a situation where ranges of densities are restricted as compared with Figure 1 in main text. For the logistic functional form, the observed pattern of recruitment shows a maximum that shifts as time progresses. This is because the densities are not high enough to reach the second compensation point that is shown in Fig. 1 of the main text; data from Jenkins et al (2008) follows this pattern. No such change is observed for the other functional forms shown in figure S5. Other sigmoid functions, such as the generalised Hyperbolic (Getz 1996) also lead to recruitment curves with more than one maximum (Fig. S6). The appearance of a compensation point at intermediate time intervals may be indicative of a sigmoid functional form of the survival probability.

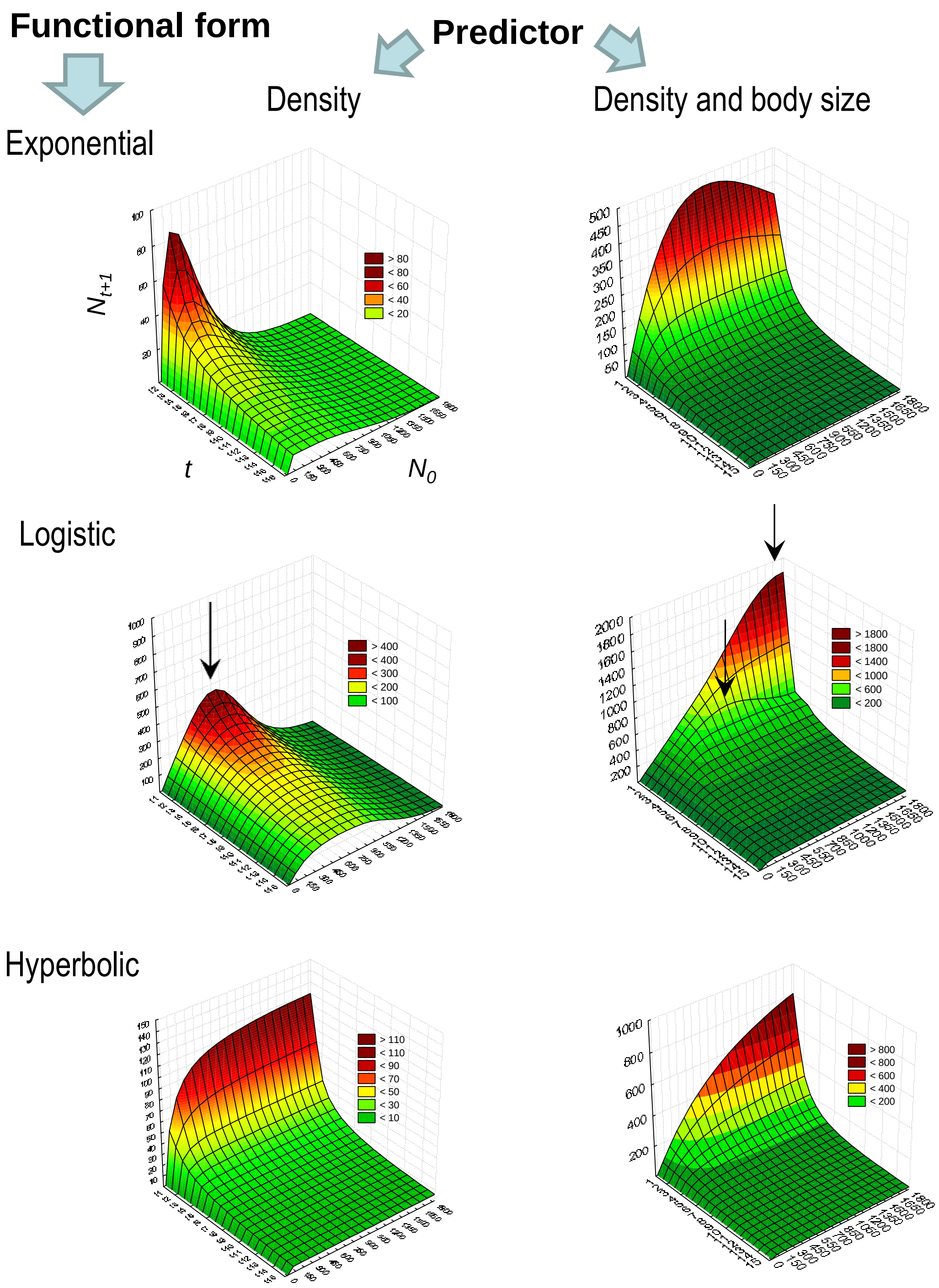

Figure S5. Models with and without body size as predictor. Arrows indicate positions of compensation points for the logistic models. In right panels, body size is modelled from the von Bertalanfy growth function defined by the growth rate (K) the initial body size (φ_0) and the asymptotic body size (φ_∞). Parameter values: Left panels: in all cases, the resource use-independent survival α = 0.95 and asymptotic size φ_∞=1. Right Panels**:** α = 0.99 and φ_∞=1 in all cases; Exponential: β=0.2, *K*=0.01, φ_0 = 0.01; Logistic: β=0.1, *K*=0.01, φ_0 = 0.01; Hyperbolic: β=0.1, *K*=0.05, φ_0 = 0.1.

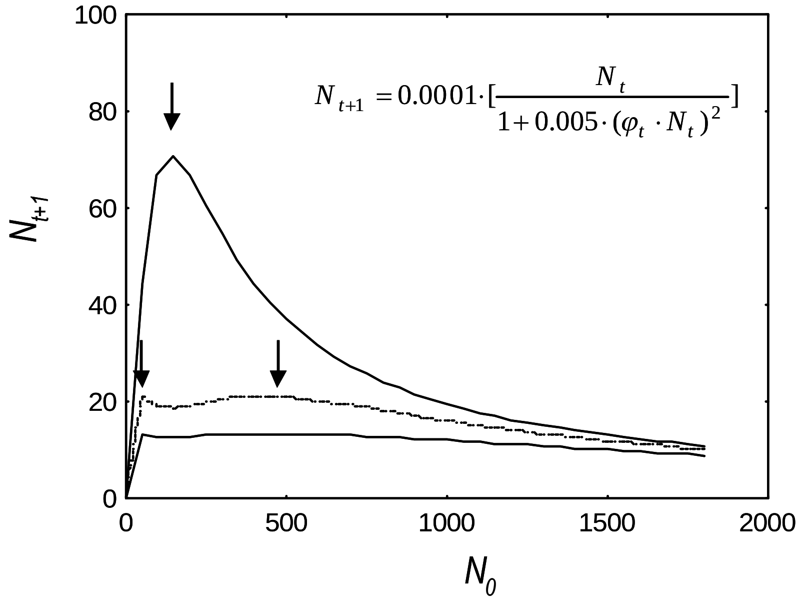

Figure S6. Recruitment model based on the generalised hyperbolic functional form including body size Parameters are as follows, φ_0 = 0.1, K=0.3, φ_{00} = 1. Arrows indicate maxima for the first and second time steps.

References:

Getz WM (1996). A hypothesis regarding the abruptness of density dependence and the growth rate of populations. Ecology 77:2014–2026.

Jenkins SR, Murua J, Burrows M T (2008) Temporal changes in the strength of density-dependent mortality and growth in intertidal barnacles. J Anim Ecol 77: 573–584.
