# Supplementary material for: Combining Traits and Density to Model Recruitment of Sessile Organisms
Source: PLoS One. 2013 Mar 1;8(3):e57849. doi: 10.1371/journal.pone.0057849 (PMC3585730; doi:10.1371/journal.pone.0057849)
Supplement: Appendix S5 — A model simulation for threshold survival. (PDF) [file pone.0057849.s005.pdf]

Appendix S5 - A model simulation for threshold survival

Here, we develop a model based on threshold survival patterns. We assume that survival chances of an individual depend on the number of individuals located in its neighbourhood. The survival remains constant = s_1 until a threshold in resource use (R_L) is reached over which survival chances drops to s_2; for simplicity s_1=1 and s_2 = 0, i.e. the there is a sharp threshold.

The model also assumes that (1) individuals are distributed as Poisson and (2) resource use responds to body size following a Von Bertalanffy growth curve. Growth is deterministic in that all individuals start with the same initial body size and grow at the same rate. This simplification means that the resource use at each time interval follows also a Poisson distribution with parameter π. This approach is valid because the objective here is to focus on the combination of threshold phenomena at the individual level and Poisson distribution of individuals.

The model is as follows:

$$N_{t+1} = \alpha \cdot N_t \cdot Cumpoiss(\pi_t)$$

$$\pi_t = \frac{N_t \cdot \varphi_t}{A}$$

$$\varphi_{t+1} = \varphi_t \cdot e^{-K} + \varphi_\infty \cdot (1 - e^{-K})$$

where α is the resource-independent parameter as in the models developed in the main text; $Cumpoiss(\pi_t)$ is the cumulative Poisson distribution evaluated between 0 and R_L and defines the fraction of organisms with a number of neighbours between 0 and R_L: this is the fraction of survivors according to the restrictions imposed by the values of s_1 and s_2. The parameter *A* is a scaling constant for surface area so that average density is defined as N_t/A. Body size is modelled according to the Ford-Walford version of the Von Bertalanffy growth curve, $\varphi_{t+1} = \varphi_t e^{-K} + \varphi_\infty (1\text{-}e^{-K})$, characterised by the growth rate K and the asymptotic body size φ_∞.

Figure S7 shows examples of simulations based on 40 sites, with and without growth. When animals grow in body size (K= 0.9) the settler-recruitment curve changes from the first to the

second time step. For the range of densities considered here, the settler recruit function is monotonic increasing in the first time step; in the subsequent steps, the curves shows a compensation point: this pattern resembles that shown by Jenkins et a. (2008), and that modelled by a logistic functional form in this paper. The case of no growth (K=0) is equivalent to a model based only on density, and it is shown to illustrate the fact that the settlement curve follows the general shape irrespective of time. Changes in the shape of the settlement-recruit function may therefore reflect threshold phenomena in individual survival in response to barnacle density and growth.

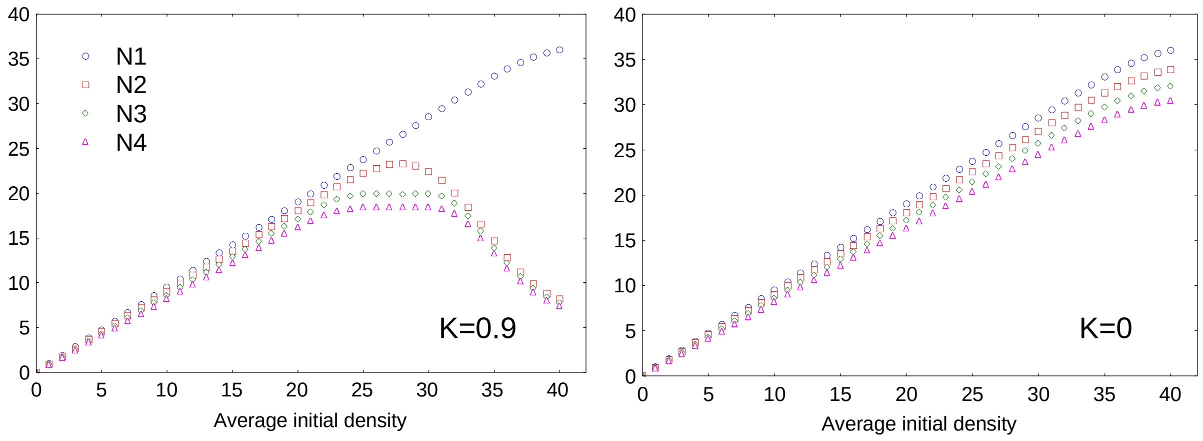

Figure S7. Model simulations of recruitment curves based on Poisson distribution of organisms and threshold individual survival. Left Panel: model with grow in body size (K=0.9); right panel: model without growth. For simplicity only the first four time steps are shown. Initial densities varied from 1 to 40, α=0.99, R_L=50, A=1, φ_0=1, φ_∞=2.
